# Natural History of Hepatic Hemangiomas Larger Than 10 cm: Imaging Findings and Clinical Course of 22 Cases

**DOI:** 10.7759/cureus.50563

**Published:** 2023-12-15

**Authors:** Yasuyuki Onishi, Tsuyoshi Ohno, Hironori Shimizu, Kotaro Shimada, Hiroyoshi Isoda, Takamichi Ishii, Atsushi Takai, Yuji Nakamoto

**Affiliations:** 1 Diagnostic Imaging and Nuclear Medicine, Kyoto University, Kyoto, JPN; 2 Surgery, Kyoto University, Kyoto, JPN; 3 Gastroenterology and Hepatology, Kyoto University, Kyoto, JPN

**Keywords:** adult, imaging findings, hemangiomatosis, natural history, liver hemangioma

## Abstract

Introduction: The natural history of a large hepatic hemangioma is important in determining the treatment strategy. Although several studies have assessed the natural history of hepatic hemangiomas, no study has focused on hepatic hemangiomas measuring >10 cm. The aim of this study was to assess the natural history of hepatic hemangiomas measuring >10 cm by evaluating imaging findings and clinical course.

Methods: Computed tomography (CT) and magnetic resonance imaging (MRI) reports at Kyoto University Hospital, Kyoto, Japan, between January 2001 and March 2023 were retrospectively searched to find adult patients with hepatic hemangiomas >10 cm. Patients who were followed up without treatment for over six months were included. The maximum diameter of the hepatic hemangioma was compared between the baseline and the final CT or MRI. The clinical course of the patients was evaluated.

Results: Twenty-two patients (17 women, five men; median age, 51 years) were identified. The median diameter of hepatic hemangiomas in the baseline study was 114 mm. Two patients had abdominal distention at the time of the baseline imaging, whereas the others were asymptomatic. After follow-up without treatment (the median; 95.5 months), enlargement, no change, shrinkage of hepatic hemangioma was observed in six, 11, and five patients, respectively. The median growth rate of hepatic hemangiomas was 2.5 mm/year. Two patients underwent liver resection for hepatic hemangioma, while the others were followed up without treatment. In four patients, symptoms appeared or worsened. Two patients died: one patient died from prostate cancer progression; the cause of death for the other was not confirmed.

Conclusion: Hepatic hemangiomas show a slow growth rate during follow-up, and shrinkage is occasionally observed. Some patients experience new symptoms or aggravation of symptoms; however, deaths associated with hepatic hemangiomas are uncommon.

## Introduction

Hepatic hemangiomas are the most common benign tumor of the liver, with an incidence of up to 20% based on autopsy studies [1]. Most hepatic hemangiomas are detected incidentally, present no signs or symptoms, and require no treatment [2,3]. Treatment is indicated when a hepatic hemangioma causes symptoms, compresses adjacent organs (gastric outlet obstruction, Budd-Chiari syndrome), ruptures into the intraperitoneal space, or causes Kasabach-Merritt syndrome [3, 4]. The size of the lesion and the growth speed of hepatic hemangiomas are considered important factors in determining the indication for surgery [4]. Some authors have argued that a hepatic hemangioma >10 cm in diameter should be called a giant hemangioma [5-7], while others used as a 10 cm diameter hepatic hemangioma as an indication for surgery [6-10]. Therefore, 10 cm can be used a cut-off value for the diameter of a hepatic hemangioma. Several studies have evaluated the natural history of hepatic hemangiomas [11-16]. However, none of these studies has focused on hepatic hemangiomas measuring >10 cm in diameter. Knowing the natural history of hepatic hemangioma >10 cm in diameter would be helpful in determining a treatment.

Hepatic hemangiomatosis is a rare condition in which the liver parenchyma is replaced with hemangiomatous lesions [17]. However, hepatic hemangiomatosis was reported to be commonly observed in patients with a large hemangioma (>8 cm) [17]. The presence and extent of hepatic hemangiomatosis affect the surgical technique of resection of a large hepatic hemangioma and are clinically important [17]. Thus, the natural history of hepatic hemangiomatosis is also important in determining a treatment for hepatic hemangioma. However, the natural history of hepatic hemangiomatosis remains largely unknown [18,19]. This study aimed to evaluate the natural history of hepatic hemangiomas >10 cm in size. In addition, this study aimed to evaluated the natural history of hepatic hemangiomatosis in patients with hepatic hemangiomas >10 cm.

## Materials and methods

Patients

Computed tomography (CT) and magnetic resonance imaging (MRI) reports at Kyoto University Hospital, Kyoto, Japan, between January 2001 and March 2023 were electronically searched for cases of hemangiomas. Adult patients (≥18 years of age) with hepatic hemangiomas with a maximum diameter of >10 cm on axial imaging were included. For each patient, the baseline study was defined as the oldest study (CT or MRI) of the upper abdomen, and the final study was defined as the latest study (CT or MRI) of the upper abdomen during follow-up in patients who did not undergo treatment, such as surgery and embolization, for hepatic hemangiomas. When a hemangioma was initially small but increased to more than 10 cm in diameter during follow-up, the first imaging study indicating the hemangioma with a maximum diameter of >10 cm was defined as the baseline study. The follow-up period was defined as the period between the baseline and the final study. Patients with a follow-up period longer than six months were included in this study. Patients with a history of hepatic hemangioma treatment were also excluded.

This retrospective study was approved by the Ethics Committee of Kyoto University Graduate School and Faculty of Medicine (approval no. R3936).

Diagnosis of hepatic hemangiomas and hepatic hemangiomatosis

The diagnosis of hepatic hemangiomas was confirmed using dynamic contrast-enhanced CT or dynamic contrast-enhanced MRI of the abdomen, which demonstrated peripheral nodular enhancement on the arterial phase, followed by centripetal filling of the lesion on the delayed phase. The presence of hepatic hemangiomatosis was evaluated using dynamic contrast-enhanced CT or fat-saturated T2-weighted MRI of the abdomen. Hemangiomatosis was defined as the presence of diffuse geographic or innumerable confluent small nodular enhancements with poorly defined margins on arterial-phase CT. The enhancement had to become more homogeneous or show filling in on delayed-phase CT. On fat-saturated T2-weighted MR, hemangiomatosis was defined as the presence of diffuse geographic or confluent innumerable high-intensity small nodular signals with poorly defined margins. Hemangiomatosis was also classified as diffuse or localized. When hemangiomatosis spread throughout the liver, it was classified as diffuse. When hemangiomatosis spared some areas of the liver, it was classified as localized.

Image evaluation

A board-certified radiologist (Y.O.) with 12 years of experience in liver imaging confirmed the diagnosis of hemangioma based on the imaging findings and evaluated the other imaging findings. The location of the hepatic hemangioma was classified as right or left. When the hemangioma was located mainly to the right of the round ligament of the liver, it was classified as right, and when it was located mainly to the left of the round ligament, it was classified as left. In addition, in the baseline study, the maximum diameter of the hemangioma was measured on axial images. In the final study, the maximum diameter of the same lesion was measured on the axial images. The maximum diameter of the hemangioma in the final study was compared with that in the baseline study, and the change in hemangioma size was classified into three groups: enlargement, >120%; no change, 80-120%; and shrinkage, <80%. The growth rate of the hepatic hemangioma was defined as ((maximum diameter on final study)-(maximum diameter on baseline study))/(follow-up period). Subsequently, the median growth rate was calculated.

The presence of hepatic hemangiomatosis was evaluated in all patients in the oldest studies (MRI or dynamic CT) of the upper abdomen. When hemangiomatosis was observed, the oldest studies (MRI or dynamic CT) and the latest studies (MRI or dynamic CT) of the upper abdomen were compared during the follow-up period. Changes in hemangiomatosis were classified as enlargement, no change, or shrinkage. When hemangiomatosis occupied more than 1.5 of the area in the latest study compared to the oldest study, it was defined as an enlargement of hemangiomatosis. Hemangiomatosis that occupied less than half of the area in the latest study compared with the oldest study was defined as shrinkage of hemangiomatosis. If the change was not classified as an enlargement or shrinkage, it was classified as no change. In patients without hepatic hemangiomatosis, the number of hepatic hemangioma was counted and classified as single or multiple.

The clinical course of each patient was checked by reviewing electronic medical records. The symptoms and signs of hepatic hemangiomas were assessed. Treatment of hepatic hemangiomas was also recorded.

CT and MRI techniques

CT images were obtained using a 4- to 320-detector row CT scanner (Aquilion, Aquilion One, Aquilion Prime; Canon Medical Systems Corporation, Japan). Our standard dynamic abdominal CT protocol included unenhanced, arterial, and delayed-phase images. The contrast agent was administered at a dose of 600 mgI/kg. A bolus-tracking system was used to obtain images at an appropriate time. The region-of-interest cursor for bolus tracking was placed over the aorta at the level of the celiac axis, and the trigger threshold was set at 200 HU. Arterial phase images were obtained 23 seconds after the trigger, and delayed-phase images were obtained 80 seconds after the trigger. Five-mm or 7-mm slice thickness images were used for image evaluation.

MRI examinations were performed using a 1.5- or 3-T system (Magnetom Avanto, Magnetom Prisma Fit, Magnetom Skyra, Magnetom Sola, and Magnetom Trio Tim, Siemens Healthcare; Genesis Signa, General Electric Medical Systems). Breath-hold fat-saturated T2-weighted fast spin-echo or turbo spin-echo sequences were obtained. Multiphase contrast-enhanced breath-hold T1-weighted gradient-echo sequences were obtained before and after contrast medium injection. Gadolinium-ethoxybenzyl-diethylenetriamine pentaacetic acid (Gd-EOB-DTPA) (Primovist, Bayer Schering Pharma, Berlin, Germany) and gadolinium-based extracellular contrast agents (Omniscan, General Electric Healthcare, Illinois, USA) were used. Gd-EOB-DTPA contained 0.25 mmol gadolinium (Gd)/mL and 0.025 mmol Gd/kg body weight was administered. Gd-based extracellular contrast agents contained 0.5 mmol Gd/mL and 0.1 mmol Gd/kg body weight was administered. All contrast agents were injected at a rate of 1 mL/s, followed by a 10 mL saline flush. A bolus-tracking technique was used to obtain images at an appropriate time. The arterial and delayed phases were obtained at 0 and 80 seconds after the detection of contrast in the abdominal aorta.

## Results

Patients

A total of 37 patients were identified. Of these, seven patients were excluded because they underwent surgery (n = 6) or transarterial embolization (n = 1), and the follow-up period was less than six months (n = 6). Furthermore, two more patients were excluded because of a previous history of treatment for hepatic hemangiomas. Thus, 22 patients (17 women and five men) comprised the study population, with a median age of 51 years at the baseline study. In all patients, dynamic contrast-enhanced CT or dynamic contrast-enhanced MRI of the liver was performed, and a diagnosis of hepatic hemangiomas was confirmed. The reasons for hepatic hemangioma detection were incidental imaging findings (n = 8), medical checkups (n = 6), evaluation of a malignant neoplasm or follow-up of a malignant neoplasm (n = 3), and not available (n = 5). None of the patients had hepatitis B or C virus infections. At the time of the baseline study, 20 patients were asymptomatic, while two patients complained of abdominal distention. At the time of the baseline study, the Child-Pugh score was five in 18 patients, six in two patients because of hypoalbuminemia, and seven in one patient because of moderate ascites. In one patient, no blood tests were performed, and the Child-Pugh score could not be calculated.

Imaging findings

**Figure 1:**
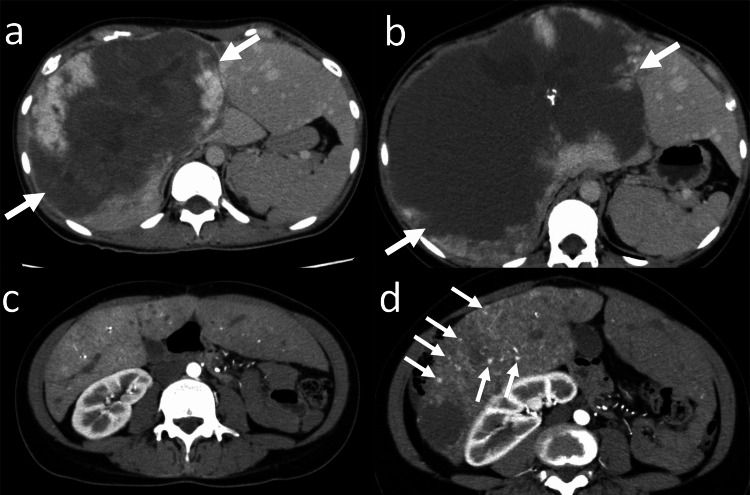
Enlargement of a hepatic hemangioma and hemangiomatosis in a 31-year-old woman (Case 1 in Table 1) (a) Axial delayed-phase computed tomography (CT) (baseline study) of the upper abdomen shows a hepatic hemangioma (arrows) with a diameter of 182 mm located in the right lobe and the medial segment of the liver. (b) Axial delayed-phase CT (final study) of the upper abdomen obtained at the 91-month follow-up shows enlargement of the hepatic hemangioma (arrows). The diameter of the hepatic hemangioma is 240 mm. (c) Axial arterial-phase CT (baseline study) at the right kidney shows no hepatic hemangiomatosis. (d) Axial arterial-phase CT (final study) at the right kidney shows multiple small foci of nodular enhancement (arrows) in the right lobe of the liver, consistent with hepatic hemangiomatosis.

**Figure 2:**
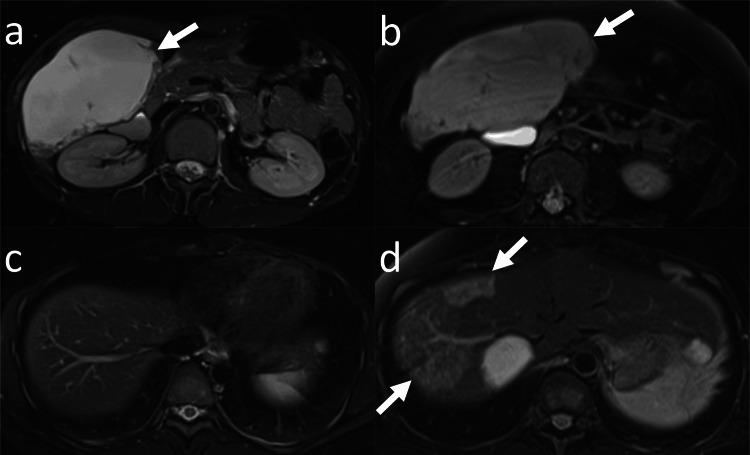
Enlargement of a hepatic hemangioma and hemangiomatosis in a 32-year-old woman (Case 2 in Table 1) (a) Axial fat-saturated T2-weighted magnetic resonance imaging (MRI) (baseline study) of the upper abdomen shows a hepatic hemangioma (arrow) with a diameter of 109 mm located in the right lobe of the liver. (b) Axial fat-saturated T2-weighted MRI (final study) of the upper abdomen obtained at the 138-month follow-up shows an enlargement of the hepatic hemangioma (arrows). The diameter of the hepatic hemangioma is 149 mm. (c) Axial fat-saturated T2-weighted MRI (baseline study) at the diaphragm shows no hepatic hemangiomatosis. (d) Axial fat-saturated T2-weighted MRI (final study) at the diaphragm shows innumerable small nodular high signal intensity (arrows), consistent with hepatic hemangiomatosis.

**Figure 3:**
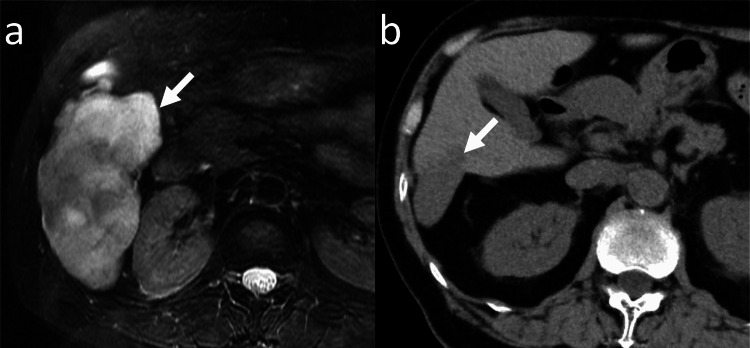
Shrinkage of a hepatic hemangioma in a 57-year-old woman (Case 15 in Table 1) (a) Axial fat-saturated T2-weigheted magnetic resonance imaging (baseline study) of the upper abdomen shows a hepatic hemangioma (arrow) with a diameter of 106 mm in the right lobe of the liver. (b) Axial unenhanced computed tomography (final study) obtained at the 188-month follow-up shows shrinkage of the hepatic hemangioma (arrow). The diameter of the hepatic hemangioma is 46 mm.

The imaging findings and clinical courses of all patients are shown in Table 1. Hepatic hemangiomas were detected on the right and left sides of the liver in 16 and six patients, respectively. The median maximum diameter of the hepatic hemangiomas at the baseline study was 114 mm (interquartile range (IQR): 103 mm to 170 mm). The median follow-up period was 95.5 months (IQR: 50 to 150 months). Enlargement, no change, and shrinkage of the hepatic hemangiomas were observed in six, 11, and five patients, respectively (Figures 1-3). The median growth rate of hepatic hemangiomas was 2.5 mm/year (IQR: −2.2 mm/year to 4.7 mm/year).

**Table 1:** Imaging findings and clinical course of patients with hepatic hemangiomas (n = 22) ⁎Size changes in hepatic hemangiomatosis are shown in parentheses in this column. ⁑Values in parentheses in this column are the periods (months) between the oldest and latest dynamic contrast-enhanced CT or MRI, with which the change in the size of hemangiomatosis was evaluated. These values are shown only in patients who had hemangiomatosis in the oldest study. Please note that the value in parenthesis is smaller than that without parenthesis because unenhanced CT was available as a baseline and final study. †This patient complained of abdominal distention at the beginning of follow-up. Symptoms did not change during follow-up. ‡In this patient, only non-enhanced CT scans were used in the follow-up study.

Cases	Age (years)/ Sex	Change in the diameter of hepatic hemangioma (mm)	Classification of size change	Hemangiomatosis	Period between baseline and final study (months)	Signs or symptoms that appeared or aggravated during follow-up	Outcome
1	31/F	182→240	Enlargement	Localized (enlargement⁎)	91 (91⁑)	Abdominal distention appeared	In follow-up
2	32/F	109→149	Enlargement	Localized (enlargement)	138 (138)	No	In follow-up
3	34/F	101→119	No change	Localized (enlargement)	66 (66)	No	Lost
4	44/F	141→162	No change	No	65	No	In follow-up
5	45/F	166→213	Enlargement	Localized (enlargement)	142 (100)	Developed abdominal distention and difficulty in moving	In follow-up
6	46/F	102→140	Enlargement	Localized (enlargement)	98 (98)	No	In follow-up
7	48/F	170→179	No change	Localized (no change)	18 (18)	No	In follow-up
8	49/F	204→175	No change	Localized (no change)	93 (38)	No†	In follow-up
9	49/F	103→125	Enlargement	No	50	No	Right lobectomy
10	51/M	221→198	No change	Diffuse (enlargement)	128 (128)	Kasabach–Merritt syndrome appeared and abdominal distention aggravated	In follow-up
11	51/F	101→58	Shrinkage	No	160	No	In follow-up
12	51/F	117→117	No change	Localized (no change)	39 (10)	No	In follow-up
13	52/F	167→67	Shrinkage	Localized (shrinkage)	211 (163)	No	In follow-up
14	53/M	105→114	No change	No	62	No	In follow-up
15	57/F	106→46	Shrinkage	No	188	No	In follow-up
16	58/F	111→126	No change	No	15	No	Right lobectomy
17	60/F	107→86	Shrinkage	Localized (no change)	150 (71)	No	In follow-up
18	61/F	103→79	Shrinkage	Localized (no change)	209 (29)	No	Follow-up was completed
19	66/M	284→350	Enlargement	Localized (enlargement)	174 (174)	Developed abdominal distention and difficulty in moving	Died from an unknown cause
20	66/F	153→137	No change	Diffuse (no change)	137 (136)	No	Follow-up was completed
21	68/M	184→171	No change	Localized (N.A. ‡)	46	No	Died due to progresssion of prostate cancer
22	71/M	102→114	No change	No	17	No	In follow-up

Hemangiomatosis was observed in 15 patients in the oldest studies: the localized form in 13 patients and the diffuse form in two patients. During the follow-up period, two or more dynamic contrast-enhanced CT or MRI studies of the abdomen were performed in 14 patients with hepatic hemangiomatosis, and changes in hepatic hemangiomatosis were evaluated. The mean interval between the oldest and latest dynamic CT or MRI was 94.5 months. Enlargement, no change, and shrinkage of hepatic hemangiomatosis were observed in seven, six, and one patient, respectively. The relationship between the change in the hepatic hemangioma size and hepatic hemangiomatosis in the 14 patients is shown in Table 2. This table suggests a tendency that hepatic hemangiomatosis enlarges in patients with a hepatic hemangioma that shows enlargement. In all patients without hepatic hemangiomatosis (n = 7), hepatic hemangiomas were multiple.

**Table 2:** Relationship between the size change of hepatic hemangioma and hepatic hemangiomatosis (n = 14)

	Hemangiomatosis		
Hemangioma	Shrinkage	No change	Enlargement
Shrinkage	1	2	0
No change	0	4	2
Enlargement	0	0	5

We evaluated the effect of age and sex on the growth rate of hepatic hemangiomas. Since the median age of the enrolled patients was 51 years, the growth rate of hepatic hemangiomas was evaluated in women aged 50 or <50 years and in those >50 years. This evaluation was not conducted in male patients as only five male participants were enrolled. Notably, for women who reached the age of 51 years during the follow-up period, growth rates were calculated for those aged 50 or <50 years and those aged >50 years; both values were used for evaluation. The median growth rate of hepatic hemangiomas in female patients aged 50 or <50 years was 4.0 mm/year and that in those aged >50 years was −2.2 mm/year.

Clinical course

Hepatic hemangiomas were treated in two patients, and both received right lobectomy. Although the two patients were asymptomatic, surgery was performed owing to the enlargement of the hepatic hemangiomas and the patient’s desire for surgical removal. In the remaining 20 patients, hepatic hemangiomas were followed without treatment. Two patients died during follow-up. A patient died from prostate cancer progression. The cause of death could not be identified in the other patient. This patient complained of abdominal distention and difficulty moving during follow-up. One of the two patients with abdominal distention at the baseline study developed a bleeding tendency during follow-up and was diagnosed with Kasabach-Merritt syndrome. The other patient showed no changes in abdominal distention. Two more patients developed symptoms during follow-up: abdominal distention in one patient and abdominal distention and difficulty moving in the other. In two patients, follow-up was discontinued because the hemangioma shrunk in size after a long follow-up period. A patient was lost during follow-up. Hepatic hemangioma rupture was not observed in any patient.

## Discussion

The median growth rate of hepatic hemangiomas was 2.5 mm/year. This result demonstrates a slow growth rate of hepatic hemangiomas and is comparable to the results of a previous study that showed a growth rate of 4.7 mm/year for hepatic hemangiomas with diameters >10 cm [13]. Spontaneous regression of hepatic hemangiomas is well known, and previous studies have reported various rates of decrease in the diameter of hepatic hemangiomas, ranging from 8.6% to 45.4% [12,13,15,16,20]. The average diameter of hepatic hemangiomas evaluated in previous studies was less than 5 cm. This study demonstrated that hepatic hemangiomas measuring >10 cm commonly shrink (22.7%). The median growth rate of hepatic hemangiomas in women aged 50 or <50 years was 4.0 mm/year and that in those aged >51 years was −2.2 mm/year. This result is in line with the results of previous studies: hepatic hemangiomas in younger female patients (<40 or 45 years) tend to enlarge, and those in older female patients (>40 or 45 years) tend to shrink [15,16]. It is advisable to determine the treatment strategy with the understanding that hepatic hemangiomas >10 cm grow at a slow rate and occasionally shrink.

Hemangiomatosis was observed in 15 patients (68.2%). Our results were consistent with those of a previous study that evaluated the imaging findings of patients with hepatic hemangiomas >8 cm and found that 44% of the patients had hepatic hemangiomatosis [17]. Changes in size in hepatic hemangiomatosis were evaluated in 14 patients. Enlargement of hepatic hemangiomatosis was observed in seven patients, and enlargement of hepatic hemangiomas was observed in five of the seven patients. The presence and extent of hepatic hemangiomatosis are important because they influence the optimal liver resection technique and the functional residual liver volume [17]. Thus, clinicians should pay attention to temporal changes not only in hepatic hemangiomas but also in hemangiomatosis, especially in patients with enlarging hepatic hemangiomas.

During follow-up, four patients experienced the appearance or aggravation of symptoms. Two patients died. One patient died from prostate cancer. In the other patient, the cause of death was not clear. Twenty patients were followed without treatment, while two patients underwent liver resection. Kasabach-Merritt syndrome developed in one patient. Considering the relatively long follow-up period (median: 95.5 months), this study demonstrates that hepatic hemangiomas with a diameter >10 cm are clinically stable for a long time, and death associated with hepatic hemangiomas is not frequent.

This study has some limitations. First, the study was conducted at a single hospital, and the study population was small. Second, this was a retrospective study, and information on symptoms of the patients’ may be incomplete. Third, the evaluation of temporal changes in hemangiomatosis was subjective and lacked objectivity.

## Conclusions

Hepatic hemangiomas >10 cm show slow growth rates and occasionally shrink during follow-up. Hepatic hemangiomatosis is commonly observed in patients with hepatic hemangiomas, and hemangiomatosis, as well as hemangioma, changes during the follow-up period. Although some patients with hepatic hemangiomas experience new symptoms or symptom aggravation, deaths associated with hepatic hemangiomas are rare.

## References

[1] Caseiro-Alves F, Brito J, Araujo AE (2007). Liver haemangioma: common and uncommon findings and how to improve the differential diagnosis. Eur Radiol.

[2] Bajenaru N, Balaban V, Săvulescu F, Campeanu I, Patrascu T (2015). Hepatic hemangioma -review. J Med Life.

[3] Leon M, Chavez L, Surani S (2020). Hepatic hemangioma: What internists need to know. World J Gastroenterol.

[4] Aziz H, Brown ZJ, Baghdadi A, Kamel IR, Pawlik TM (2022). A comprehensive review of hepatic hemangioma management. J Gastrointest Surg.

[5] Toro A, Mahfouz A-E, Ardiri A (2014). What is changing in indications and treatment of hepatic hemangiomas. A review. Annals of Hepatology.

[6] Di Carlo I, Koshy R, Al Mudares S, Ardiri A, Bertino G, Toro A (2016). Giant cavernous liver hemangiomas: is it the time to change the size categories?. Hepatobiliary Pancreat Dis Int.

[7] Xie QS, Chen ZX, Zhao YJ, Gu H, Geng XP, Liu FB (2021). Outcomes of surgery for giant hepatic hemangioma. BMC Surg.

[8] Popescu I, Ciurea S, Brasoveanu V, Hrehoret D, Boeti P, Georgescu S, Tulbure D (2001). Liver hemangioma revisited: current surgical indications, technical aspects, results. Hepatogastroenterology.

[9] Abdel Wahab M, El Nakeeb A, Ali MA (2018). Surgical management of giant hepatic hemangioma: single center's experience with 144 patients. J Gastrointest Surg.

[10] Marrero JA, Ahn J, Rajender Reddy K (2014). ACG clinical guideline: the diagnosis and management of focal liver lesions. Am J Gastroenterol.

[11] Gibney RG, Hendin AP, Cooperberg PL (1987). Sonographically detected hepatic hemangiomas: absence of change over time. AJR Am J Roentgenol.

[12] Hasan HY, Hinshaw JL, Borman EJ, Gegios A, Leverson G, Winslow ER (2014). Assessing normal growth of hepatic hemangiomas during long-term follow-up. JAMA Surg.

[13] Jing L, Liang H, Caifeng L, Jianjun Y, Feng X, Mengchao W, Yiqun Y (2016). New recognition of the natural history and growth pattern of hepatic hemangioma in adults. Hepatol Res.

[14] Choi J, Yu J, Cho E, Kim J, Chung J (2018). Hepatic cavernous hemangiomas: long-term (> 5 years) follow-up changes on contrast-enhanced dynamic computed tomography or magnetic resonance imaging and determinant factors of the size change. Radiol Med.

[15] Wang A, Deng J, Qian B (2019). Natural history of hepatic hemangioma: a follow-up analysis of 534 patients. Frontiers in Life Science.

[16] Mogahed MM, Zytoon AA, Essa B, Abdellatif W, Ghanem N, ElWakeel B (2020). Natural history of hepatic hemangiomas as a guide for surgical indication. Egypt Liver J.

[17] Jhaveri KS, Vlachou PA, Guindi M, Fischer S, Khalili K, Cleary SP, Ayyappan AP (2011). Association of hepatic hemangiomatosis with giant cavernous hemangioma in the adult population: prevalence, imaging appearance, and relevance. AJR Am J Roentgenol.

[18] Liu MC, Little EC (2018). Isolated hepatic hemangiomatosis in 2 septuagenarians. Radiol Case Rep.

[19] He S, Chen W, Yang Y, Tang X, Zhou G, Zhou J, Wu C (2022). Adult diffuse hepatic hemangiomatosis: a case report and review of the literature. Clin Res Hepatol Gastroenterol.

[20] Aydin O, Acunaş B, Poyanli A, Serin KR, İbiş C, Özden İ (2021). Spontaneous regression of liver hemangiomas: a single-institution analysis of 46 patients. Eur J Gastroenterol Hepatol.

